# Mental Health in Cypriot Citizens of the Rural Health Centre Kofinou

**DOI:** 10.3390/healthcare4040081

**Published:** 2016-11-01

**Authors:** Georgios Stavrou, Lefkios Paikousis, Eleni Jelastopulu, Georgios Charalambous

**Affiliations:** 1 Department of Pharmacy, Rural Health Centre Kofinou, Larnaca 7735, Cyprus; 2 Health Management, Frederick University, Nicosia 1036, Cyprus; 3 Independent Researcher, Nicosia 1048, Cyprus; lefkiospaik@yahoo.co.uk; 4 Department of Public Health, Medical School, University of Patras, Patras 26500, Greece; jelasto@upatras.gr; 5 Department of Emergency Medicine, General Hospital of Athens “Hippocration”, Athens 11527, Greece; drcharalambous@yahoo.gr

**Keywords:** depression, anxiety, emotional distress, HADS, GDS, Health Centre

## Abstract

*Objective*: The main purpose of this study was to investigate the mental health of Cypriot citizens living in the current difficult period of economic recession. The specific objective was to investigate the different factors (gender, age, socio-economic factors, etc.) that may affect the levels of emotional distress, anxiety, and depression in patients attending the Rural Health Centre of Kofinou. *Materials and Methods*: The sample consisted of a total of 300 Cypriots who visited Kofinou Health Centre in the period between July and September 2015. For the middle-aged citizens, the Greek version of the Hospital Anxiety Depression Scale (HADS) was applied to 150 persons [1], while for the visiting senior citizens (aged over 65 years), the Greek version of the Geriatric Depression Scale (GDS) was used [2]. *Results*: *HADS:* A total of 150 people of average age 47 ± 11.5 years (min 23–max 64) participated in the study. Fifty-six percent were women. Seventy-seven percent stated they had a reduction in income (mean reduction 35% ± 25%) and 46.7% suffered from chronic disease. The 36.6% and 28.7% of the visitors showed moderate or severe forms of anxiety and depression, accordingly. Higher emotional distress is associated with lower educational level (b = −2.63, *p* < 0.001), lower income (b = −1.07, *p* = 0.017), and the presence of a chronic disease (b = 5.45, *p* < 0.001). The same factors are significantly associated with higher anxiety (Education: b = −1.20, *p* = 0.003; Income: b = −0.64, *p* = 0.01; Chronic disease: b = 2.82, *p* = 0.001). Additionally, a reduction in income (>35%) is associated with increased depression (*p* = 0.028). *GDS:* 150 patients out of which 77 were women (51.3%). The average age of participants was 72 ± 5.5 years. Ninety-three (62%) participants declared a reduction in income due to the financial crisis (mean reduction 20% ± 8%), while 139 (92.7%) stated that they had chronic disease. Fifty-three participants (35.3%) thought they had symptoms of depression after the economic crisis. The women showed higher level of geriatric depression symptoms than men (b = −1.96, *p* = 0.005), while age is associated with higher levels of GDS (b = 0.16, *p* = 0.006). *Conclusions*: The study shows that stress levels, depression, and emotional distress are increased in specific population groups. The main variables associated with the mental health of the participants are the presence of a chronic disease, income, and level of education.

## 1. Introduction

It is well known and commonly accepted that issues related to mental health are directly and indirectly caused by deprivation, poverty, inequality, and many other social and financial factors. Therefore, economic recessions, which have been recorded, are still being historically documented, showing that the world population has high-risk periods in terms of mental health, even up to this date [3].

The economic recession that occurred in 2007 in the U.S. grew to involve the rest of the world and had a significant impact on the European Union (EU) and more specifically on Cyprus. This negative impact has led Cyprus’s economy to a crucial decline, thus having a dramatic rise of the unemployment rates and leading a large number of people to live under poor conditions [4]. Simultaneously, the increase in the national debt, the agreements of the country’s financial assistance facility, as well as the loan agreements have led Cyprus to apply restrictive measures and severe cuts in public spending, even within the health sector and welfare services.

The rising unemployment, reduced incomes, and the living conditions of the citizens have increased the risk of living in poverty and underprivileged conditions. Thus, citizens and predominantly young people are inevitably affected. Young people have become very anxious and fearful for the future [5,6]. Those same conditions also lead to suicide attempts, especially in the elderly population. Unemployment, poverty, stress, and insecurity are therefore risk factors, which constantly increase the rates of depression, therefore negatively affecting people across all ages [7].

According to the World Health Organization (WHO), depression is a universal mental disorder characterized by sadness, loss of interest or pleasure, feelings of guilt or low self-esteem, sleep deprivation or lack of appetite, and fatigue and inability to concentrate [8]. Furthermore, based on research, depression often appears at a young age and mostly affects the female population. It is important to mention that citizens who have either lost their jobs due to adverse economic conditions or have been generally made unemployed are at a higher risk of developing symptoms of anxiety and depression [9].

As a consequence of the social perceptions and attitudes of Cypriot citizens, many cases remain either undiagnosed or are not adequately addressed in the Cypriot community [4]. There is a large proportion of patients presenting with depressive syndromes who do not seek specialist psychiatric attention. Instead, they resort to traditional forms of counselling or palliative assistance, while others passively anticipate in recovering the symptoms. There are also many people visiting primary medical care services mainly for various psychosomatic symptoms. Nevertheless, clinical depression remains sub-optimally diagnosed [10].

In conclusion, this current research focuses on exploring the mental health of Cypriot citizens in the area of Kofinou Rural Health Centre (RHC) along with the various factors affecting it. Due to the lack of research in this field, it is recommended that more studies on the topic of economic crisis and its effects on the Cypriot society be carried out. It is an essential and current issue, which undoubtedly concerns not only the health services and health professionals, but also all Cypriot citizens.

## 2. Materials and Methodology

The study was cross-sectional and was carried out in the Rural Health Centre of Kofinou using two scales for estimating the mental health of patients visiting the center: the GDS and HADS, both screening tests where higher total scores indicate higher depression or anxiety symptomatology. The GDS questionnaire was given to the elderly, those over 65 years old, along with our constant guidance because of their advanced age and for improved elaboration, whereas the HADS was given to adults between 18 and 65 years old. Although HADS could be administered to both groups of patients, due to the poor education level of the elderly patients (>65) visiting the health centers in Cyprus, GDS was preferred due to the simple “yes/no” structure of the responses. All respondents were asked to self-report the percentage reduction in their income. The participation of the respondents was strictly anonymous and highly respected while giving them the option to voluntarily participate in the survey.

The study’s sample consisted of 300 adults, 150 adults over 18 years old to 65 (HADS Scale), and 150 elderly people aged over 65 (GDS Scale). Participants were patients who visited the RHC Kofinou during the period from July to September 2015 and were selected with a random sampling method.

### Statistical Analysis

Demographic characteristics and total scale scores are presented as frequency (N) and proportion (%) for categorical variables (i.e., gender, education level, anxiety and depression levels, etc.) and mean ± standard deviation for the continuous variables (i.e., age and total scale scores). Multivariate analysis for the adjusted effect of the demographic factors was conducted using linear regression models on total emotional distress, level of stress, and level of depression for the HADS scale and on total depression score for the GDS scale. The factor reduction in earnings was also included in the multivariate analysis and was determined by the investigators at 35% for the HADS scale and at 20% for the GDS scale, as these cutoffs represent the mean self-reported reduction level in the two samples, respectively. All analyses were performed with the social science analysis package SPSS (IBM Corp. Released 2012. IBM SPSS Statistics for Windows, Version 21.0. Armonk, NY, USA).

## 3. Results

### 3.1. Sample Characteristics

The study based on the HADS scale involved 150 patients; 84 (56%) were female. The mean age of the participants was 47 ± 11.5 years, with a minimum age of 23 years and a maximum of 64. The study based on the GDS scale sampled another 150 patients. Seventy-seven (51.3%) were women. The average age of participants is 72 ± 5.5 years old, with a minimum and a maximum age of 66 and 90 years old, respectively. Fifty-three (35.3%) participants believed they showed signs of depression after the economic crisis (Table 1). The demographic and clinical characteristics of both samples are shown in Table 1.

### 3.2. HADS Scale

The overall HADS scale (total emotional distress) and sub-factors anxiety and depression showed excellent internal consistency index (Cronbach’s alpha = 0.921, 0.821, 0.813 respectively).

The average total HADS scale was 16.8 ± 8.9, with a possible maximum of 42. The average level in the subscales anxiety and depression was 9 ± 4.8 and 7.8 ± 4.5, respectively, with a possible maximum of 21. As shown in Table 2, 43.3% of the research’s participants have normal stress levels and 50% normal depression levels.

### 3.3. HADS Multivariate Analysis

There were three linear regression models (adjusted demographically) on the following dependent variables: emotional distress (total scale score), level of stress, and level of depression (Table 3).

The possible range of HADS anxiety and HADS depression is 0–21; for emotional distress, 0–42. The regression analysis showed that patients with chronic disease have higher anxiety levels by 2.82 units (b = 2.82, *p* = 0.001), depression by 2.63 units (b = 2.63, *p* = 0.001), and emotional distress by 5.45 units (b = 5.45, *p* < 0.001).

Moreover, an increase in the annual income category is associated with an average of 0.64 units less in the level of anxiety (b = −0.64, *p* = 0.01) and 2.63 units less in the emotional distress level (b = −2.63, *p* < 0.001).

Every additional level of education is associated with an average of 2.63 units less in the level of emotional distress (b = −2.63, *p* < 0.001), 1.18 units less in the anxiety level (b = −1.196, *p* = 0.003), and 1.44 units less in the depression level (b = −1.436, *p* < 0.001).

Finally, a reduction in income in excess of 35% is associated with an increase in the depression levels by 1.74 units (b = 1.74, *p* = 0.028) in the HADS-depression sub-scale.

Further investigation revealed that a reduction in income in the excess of the mean reduction level of 35% is moderately associated with a lower annual income category (x^2^ = 9.027, *p* = 0.032). People in the lower income categories reported a more significant reduction in their annual income than in the higher income categories.

### 3.4. GDS Scale

The overall GDS scale showed an adequate internal consistency index (Cronbach’s alpha = 0.691). The average geriatric depression of the 150 participants was 5.77 ± 4, with an observed minimum and maximum 0 and 15, correspondingly.

#### Reference to Depression Symptoms

Based on the questionnaires, 35.3% (n = 53/150) of the participants reported that the economic crisis was the provoking factor of their depressive symptoms.

The findings revealed that 29% of the participants denied having depression, although they fulfilled the diagnostic criteria of the syndrome according to the GDS scale (i.e., GDS ≥ 7). In 71% of cases, the responses coincided with the GDS scale. In other words, the respondents denied having depression, and this was supported by the scale findings.

**Figure 1:**
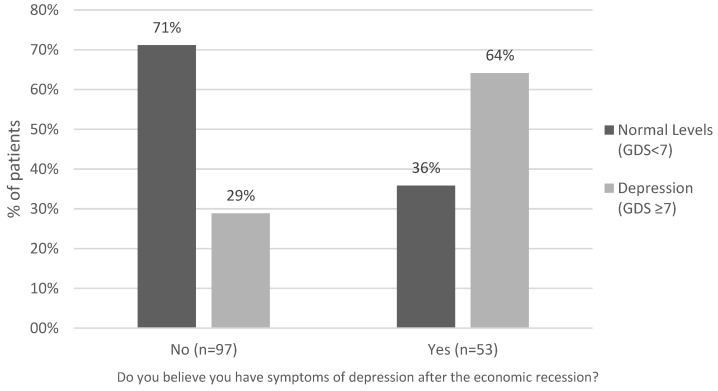
Self-reported depression symptoms vs. depression according to the GDS Scale (≥7).

According to the GDS, a similar phenomenon occurs in people who initially reported having symptoms of depression, while 36% of these individuals are ultimately not depressed (Figure 1).

### 3.5. GDS Multivariate Analysis

A linear regression model was applied to adjust the effect of the demographic characteristics in the participants’ total GDS score.

The regression analysis (Table 4) shows that men have lower levels of geriatric depressive symptoms than women by almost 2 points on average on the GDS scale (b = −1.96, *p* = 0.005). Additionally, for every additional year in their age, elderly patients have higher depressive symptoms by 0.16 points on average on the GDS (b = 0.16, *p* = 0.006). Education, the presence of chronic disease, and the reduction in the people’s salaries beyond the mean reduction level of 20% do not seem to be associated substantially with the level of geriatric depressive symptoms. It is also observed that annual income is not statistically associated with the level of geriatric depression. This is probably due to the high association of gender and income, as women in this sample are 1.92 times more likely to belong to the lowest income category of below €8000 (n = 67%–87%) than men (n = 33%–45%) (x^2^ = 32.7, *p* < 0.001).

## 4. Discussion

Our findings are supported by two different studies [11,12], which delineate the important association between gender and mental health.

In addition, Marmot et al. points out that people with lower social status are twice as likely prone to mental disorders [13]. This finding agrees with the results of this study, as demonstrated by the educational level factor. Precisely, it was illustrated that the more educated the citizen is, the less anxiety, depression, and general emotional distress they may experience [14].

Additionally, the key factor of the economic recession is the annual income decrease for each individual. Giotakos believes that reduced income, reduced labor specialization, and social alienation have shown to be associated with negative effects on physical, emotional, and psychological health, as well as an increased risk of low life expectancy [9]. His views also reinforce investigations carried out by Economou et al., which correlated unemployment rates with causes of death (studied six causes of death) at a very high percentage (83.33%) [15]. Within the same survey, it was also indicated that the patient’s financial well-being is associated with less anxiety, depression, and emotional distress.

Falagas et al. demonstrated the significant relationship between chronic disease and mental health [16]. The patients who suffer from chronic diseases tend to have higher levels of anxiety, depression, and general symptoms of emotional distress compared with non-patients.

Methodological weaknesses that emerged during the course of this investigation were as follows:

(a) Appropriate comprehension of the questionnaires by the participants, especially in cases where the patients were uneducated, could not be guaranteed. Senior citizens required guidance throughout the process, either because of a low educational level or because of a physical disability.
(b) This is a cross section of data and thus we cannot interpret any of our findings as causal or generalize for the whole population. It is vital to obtain information from the general population, as this study is limited to people who sought medical help.

## 5. Future Recommendations

Several researchers within the health sector have recognized the importance of a human’s mental health and well-being. Mental illness has been a very frequent phenomenon in the Cypriot community in the last few years. However, few studies, if any, have dealt with this crucial issue. Furthermore, as this is a cross-sectional study and no causal inferences can be made on the associations that were identified, further investigation on the drivers of emotional distress to the population should be performed, and additional concurrent stressors that could explain the depression or anxiety levels should be explored.

### 5.1. Political Authority

Each country’s health sector is undoubtedly a foremost issue that needs exceptional attention. Politicians must address the whole situation with interest by setting future goals, hence dealing and solving problems that may arise. Unfortunately, the health system in Cyprus has many weaknesses, which come at the expense of the country’s citizens. An important function of the state is the lack of political will. The state ought to set laws in order to better address such distressing situations by carrying out administrative procedures as soon as possible with the aim to better resolve arising issues.

### 5.2. Health Practitioners

In an effort to fight mental illness, health professionals need to form a group that will be responsible for the prevention of mental disease and public information. They can also organize seminars to sensitize the Cypriot community and raise awareness of the severity of this situation. Furthermore, various stereotypes and prejudices regarding depression syndromes must be gradually eliminated from Cypriot society.

## 6. Conclusions

The study shows that stress levels, depression, and emotional distress are increased in specific population groups. The main variables associated with the mental health of the participants are the presence of chronic disease, income, and level of education. There are also strong indications that reductions in earnings might be an additional stress factor.

## Figures and Tables

**Table 1:** Demographic and clinical characteristics of the sample that completed the Hospital Anxiety Depression Scale (HADS) (n = 150) and Geriatric Depression Scale (GDS) (n = 150) scale.

Variables		HADS Scale		GDS Scale	
		n	%	n	%
Gender	Woman	84	56.0%	77	51.3%
	Man	66	44.0%	73	48.7%
Age		47 ± 11.5 years/MIN = 23, MAX = 64		72.9 ± 5.5 years/MIN = 66, MAX = 90	
Education level	None	0	0.0%	11	7.3%
	Primary School	32	21.3%	99	66.0%
	Secondary	12	8.0%	17	11.3%
	Lyceum	63	42.0%	19	12.7%
	Higher Education	35	23.3%	4	2.7%
	Master/PhD	8	5.3%	0	0.0%
Annual income	Unemployed	15	10.0%	0	0.0%
	Until 8000	46	30.7%	100	66.7%
	8001–12,000	21	14.0%	30	20.0%
	12,001–18,000	25	16.7%	13	8.7%
	18,001–30,000	26	17.3%	6	4.0%
	30,001 and over	17	11.3%	1	0.7%
Reduction of the annual income due to economic crisis in the last year		116	77.3%	93	62.0%
Presence of chronic disease		70	46.7%	139	92.7%
Blood hypertension		42	60.0%	100	66.7%
Diabetes		22	31.4%	58	38.7%
Lipid disorder		28	40.0%	73	48.7%
Autoimmune disease		0	0.0%	1	0.7%
Heart disease		7	10.0%	32	21.3%
Other		12	8.0%	4	2.7%
Do you believe you have symptoms of depression after the economic recession?		-	-	53	35.3%

**Table 2:** Level of anxiety and depression-HADS (N = 150).

Variables	Anxiety Level		Depression Level	
	n	%	n	%
Normal Level	65	43.3%	75	50.0%
Mild Level	30	20.0%	32	21.3%
Moderate Level	35	23.3%	31	20.7%
Severe Level	20	13.3%	12	8.0%
Total	150	100%	150	100.0%

Normal levels (total score 0–7), Mild (8–10), Moderate (11–14), Severe (15–21).

**Table 3:** Regression analyses of emotional distress, anxiety, and depression on the socioeconomic factors.

Dependent Variable	HADS-Total Score (Emotional Distress)		HADS-Anxiety		HADS-Depression	
Predictors	b *	*p*	b *	*p*	b *	*p*
(Constant)	25.6	<0.001	14.098	<0.001	11.502	<0.001
Age	−0.059	0.445	−0.039	0.355	−0.019	0.621
Gender (women)	−2.058	0.138	−1.296	0.094	−0.762	0.274
Chronic disease	5.449	<0.001	2.817	0.001	2.632	0.001
Annual Income	−1.066	0.017	−0.642	0.01	−0.425	0.058
Education Level	−2.633	<0.001	−1.196	0.003	−1.436	<0.001
Reduction in earnings (>35%)	2.917	0.063	1.177	0.178	1.741	0.028
Model fit indices						
F (*p* value)	11.309 (<0.001)		8.986 (<0.001)		11.696 (<0.001)	
Adjusted R^2^	0.293		0.243		0.301	

* Unstandardized beta weights.

**Table 4:** Regression analyses of total GDS score on the socioeconomic factors.

Variables	b	SE	Standardised b	t	*p*
(Constant)	−5.338	4.505		−1.185	0.238
Gender (female)	−1.958	0.687	−0.248	−2.849	0.005
Age	0.161	0.058	0.223	2.778	0.006
Annual income:	−0.703	0.39	−0.153	−1.801	0.074
Chromic disease	1.279	1.149	0.084	1.113	0.267
Reduction in earnings (>20%)	0.739	0.665	0.086	1.112	0.268

Note: SE = Standard Error; Dependent variable: Total GDS score, F = 6.552, *p* < 0.001, Adj R^2^ = 0.17.
